# DNA copy number, including telomeres and mitochondria, assayed using next-generation sequencing

**DOI:** 10.1186/1471-2164-11-244

**Published:** 2010-04-16

**Authors:** John C Castle, Matthew Biery, Heather Bouzek, Tao Xie, Ronghua Chen, Kira Misura, Stuart Jackson, Christopher D Armour, Jason M Johnson, Carol A Rohl, Christopher K Raymond

**Affiliations:** 1 Rosetta Inpharmatics LLC, a wholly owned subsidiary of Merck & Co., Inc., Seattle, Washington 98109, USA; 2 Institute for Translational Oncology and Immunology (TrOn), Mainz, Germany; 3 NuGEN Technologies, Inc., Seattle, Washington, USA; 4 University of Washington, Seattle, Washington, USA; 5 Pfizer, Inc., San Diego, California, USA; 6 Merck Research Laboratories, Boston, Massachusetts, USA; 7 Amgen, Inc., Seattle, Washington, USA

## Abstract

**Background:**

DNA copy number variations occur within populations and aberrations can cause disease. We sought to develop an improved lab-automatable, cost-efficient, accurate platform to profile DNA copy number.

**Results:**

We developed a sequencing-based assay of nuclear, mitochondrial, and telomeric DNA copy number that draws on the unbiased nature of next-generation sequencing and incorporates techniques developed for RNA expression profiling. To demonstrate this platform, we assayed UMC-11 cells using 5 million 33 nt reads and found tremendous copy number variation, including regions of single and homogeneous deletions and amplifications to 29 copies; 5 times more mitochondria and 4 times less telomeric sequence than a pool of non-diseased, blood-derived DNA; and that UMC-11 was derived from a male individual.

**Conclusion:**

The described assay outputs absolute copy number, outputs an error estimate (p-value), and is more accurate than array-based platforms at high copy number. The platform enables profiling of mitochondrial levels and telomeric length. The assay is lab-automatable and has a genomic resolution and cost that are tunable based on the number of sequence reads.

## Background

DNA copy number variations occur within populations and aberrations can cause tumors, be used for drug target identification, and be used as biomarkers of tumor drug response. EGFR (epidermal growth factor receptor) amplification, for instance, is a marker for gefitinib treatment [1] and TYMS (thymidylate synthase) amplification conveys 5-fluorouracil resistance in colon tumors [2].

The next-generation sequence enables generation of millions of short sequence tags in a single experiment. Using DNA as an input, the technology has been used to resequence entire genomes, including from normal individuals [3] and from cancerous cells [4], and to resequence targeted genomic regions, such as resequencing protein coding regions to discover somatic mutations [5]. Alternatively, using RNA as an input, the technology has been used to profile RNA expression levels, where the number of sequence reads "tagging" an RNA transcript is a measure of its expression [6].

Combining these ideas and building on previous methods [7-10], this report describes the development of a sequencing-based platform to profile "expression" of DNA, inputting DNA and analyzing the resultant data using algorithms previously established for gene expression profiling. The platform resolution and cost are tunable; is lab automatable; can be used for both discovery of novel and profiling of known DNA copy number variations; outputs copy number and uncertainty as opposed to ratios; and can be used to profile mitochondria and telomeric DNA.

## Results

### Application 1: Assaying nuclear genomic copy number

We assayed the genomic copy number in a pool of DNA derived from blood from non-diseased males; a pool of DNA derived from blood from non-diseased females; and DNA from UMC-11 cells, a lung carcinoid-derived cell line. We generated and sequenced each library, aligned resultant reads to the genome, and selected reads aligning to only one genomic location (Methods).

**Figure 1:**
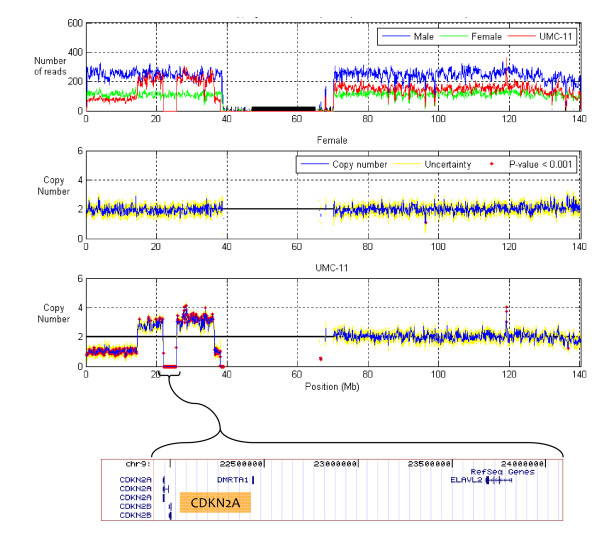
**Copy number across chromosome 9 in the male pool, female pool and UMC-11 cells**. Top: The number of sequence reads mapping to 150 kb windows, stepping at 75 kb. Second: Copy number in the female pool. Blue line, copy number; yellow lines, upper and lower bounds; red points, windows with copy number variation (p-value < 0.001). Third: Copy number in the UMC-11 cells. Bottom: Transcripts/genes in the deleted region.

Across chromosome 9, the number of reads from the male pool mapping to each genomic block is near 250 (Figure 1A, blue). From the female pool, the number of reads mapping to each block is similarly constant, at roughly 150 (Figure 1A, green). We tested normalization by the GC content in each DNA block [9,10]; however, we found that the relative number of reads mapping to a block depended more strongly on molecular biology protocols. Instead, we developed and applied a novel normalization method using the male pool as a reference, and with this normalization were able to derive the copy number, upper and lower bounds, and the significance of any deviation (Methods). In the female DNA pool, the copy number is two across the entire chromosome, with a single exception at position ~95 Mb (Figure 1B). Use of a single reference sample, processed using the same bench protocol as used for the non-reference samples, therefore eliminates the need to normalize for GC content and for sequence uniqueness within each block.

Conversely, UMC-11 cells show dramatic copy number variations. Before normalization, the number of reads mapping to each block shows many discrete levels, including blocks with no reads and blocks with roughly 75, 150, and 225 reads (Figure 1A, red). After normalization, these discrete levels map to copy number of zero, one, two, three, and four (Figure 1C). Intriguingly, the homozygously deleted region spanning from 21 to 24 Mb includes the putative tumor suppressor CDKN2A [11] (Figure 1D).

**Figure 2:**
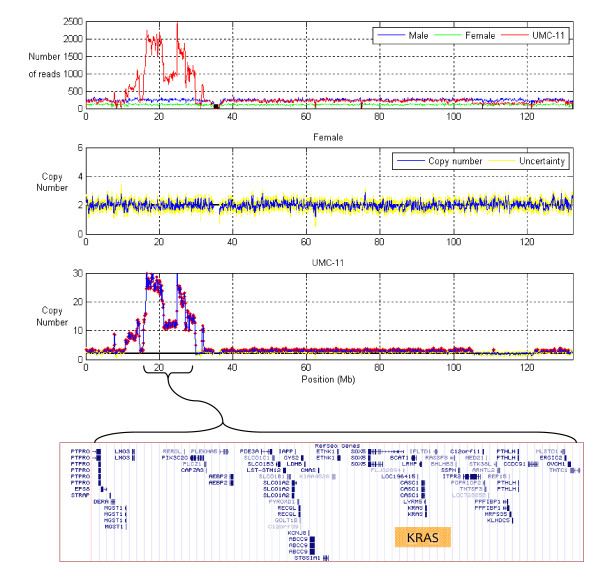
**Copy number across chromosome 12, as per Figure 1**.

Across chromosome 12, the female pool has copy number two across the entire chromosome, as expected (Figure 2B). The UMC-11 cell line is highly aberrant: copy number starts at three; is amplified to 9; falls to three; is amplified up to 29 copies; drops to three; falls to two; has a small segment at three copies; back to two; and then back to three. Again intriguingly, the highly amplified region includes the tumor-associated gene KRAS [12].

**Figure 3:**
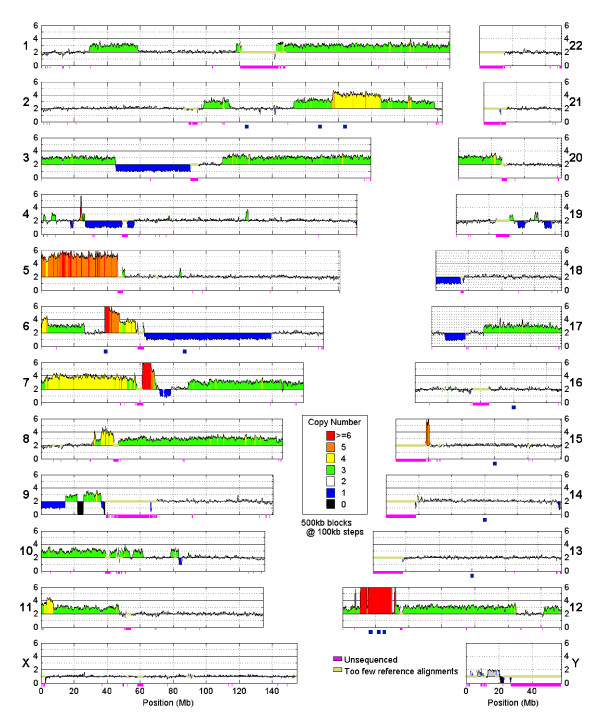
**Genome-wide copy number in the UMC-11 cells**. Copy number is assayed in 500 kb windows, stepping at 100 kb. Blocks are assigned color based on the nearest integer. Tan regions contain fewer than 50 uniquely aligning reads in the reference sample. Below each chromosome, pink marks unsequenced regions and the 12 blue boxes mark the locations assayed by qPCR.

This assay provides a genome-wide view of copy number in a single assay, allowing the full extent of copy number within a sample to be assessed (Figure 3). While chromosomes 13, 16, 21, 22, and X in UMC-11 cells show little disruption, many chromosomes show amazing copy number variation. Almost 50 Mb of chromosome 5 is present at 5 copies. An 80 Mb block of chromosome 6 is present at one copy. Many changes in copy number occur close to or across centromeres. We also identify that the cell line is derived from a male patient as chromosome X is present at one copy and many reads map to chromosome Y.

**Figure 4:**
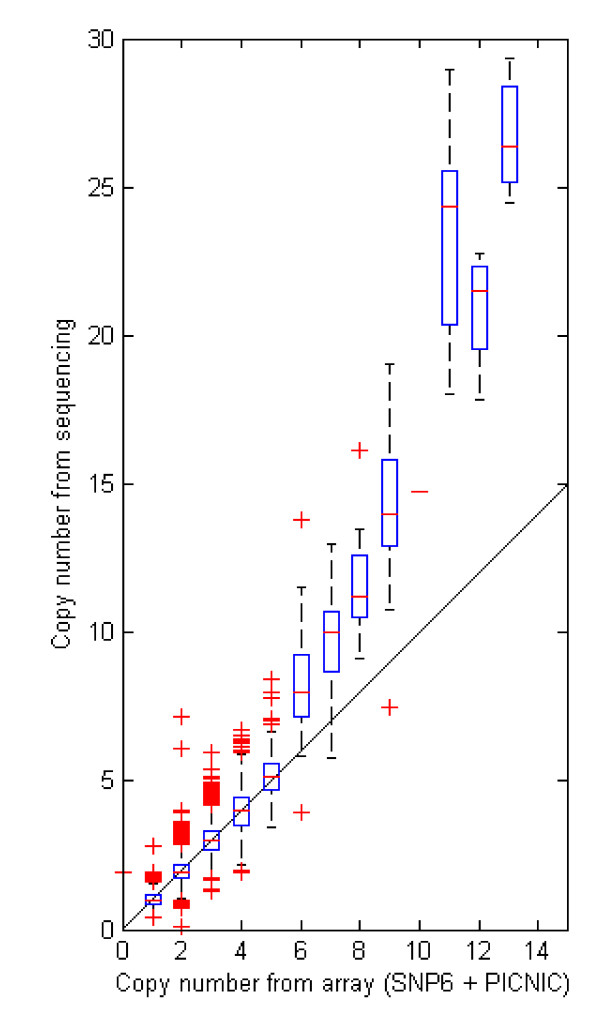
**Comparison between UMC-11 copy number from sequencing platform with that from Affymetrix SNP6 arrays analyzed using the Sanger PICNIC algorithm**.

To validate findings, we assayed copy number with qPCR at 12 locations (Figure 3, blue boxes; Table 1, Methods). The five locations predicted to have copy number two by sequencing indeed had copy number two as measured by qPCR. Copy number measurements of 1, 3, 4, and 12 also validated. At high copy number, sequencing and qPCR measurements were similar: 10 and 11; 26 and 24; 25 and 29, respectively. These results also show high correspondence with findings from an array-based platform [7] (Table 1, Additional files 1 and 2: Figures S1 and S2), including the chromosome 9 deletion near 22 Mb and the small amplification near position 120 Mb. Results from the highly amplified region of chromosome 12 qualitatively agree; however, the array-based method predicts only 13 copy number in the region where the sequencing and qPCR assays measure over 25 copy number (Table 1). Across the entire genome, the array-based data for UMC-11 cells, downloaded from the Sanger Center and analyzed with the PICNIC algorithm [13], show high concordance at copy number five and below, but discrepancies at higher copy number (Figure 4). The array-platform returns lower copy numbers than found by qPCR and sequencing, potentially the result of microarray probe saturation [8].

**Table 1:** Validation of sequencing copy number assayed by qPCR.

Chromosome	Coordinate	qPCR	Sequencing	Array
2	124,585,349	2	2	2
2	168,899,639	3	3	3
2	184,059,790	4	4	4
6	39,100,000	11	10	7
6	87,009,800	1	1	1
12	16,995,694	24	26	13
12	21,762,192	12	12	8
12	25,206,027	29	25	11
13	60,000,100	2	2	2
14	60,000,100	2	2	2
15	60,000,100	2	2	2
16	60,000,100	2	2	2

Values are rounded to the nearest integer.

### Application 2: Assaying genetic loci copy number

Conceptually, one can define biological elements in terms of gene loci rather than genomic blocks, and thus, by counting the number of reads uniquely mapping to each locus, generate a gene-copy number table. Here, we defined locus coordinates as the greater of the transcript start-to-stop span or 60 kb centered on the loci. As before, we normalized the counts to the reference male pool, assuming the male pool is diploid across autosomes and haploid across allosomes.

**Figure 5:**
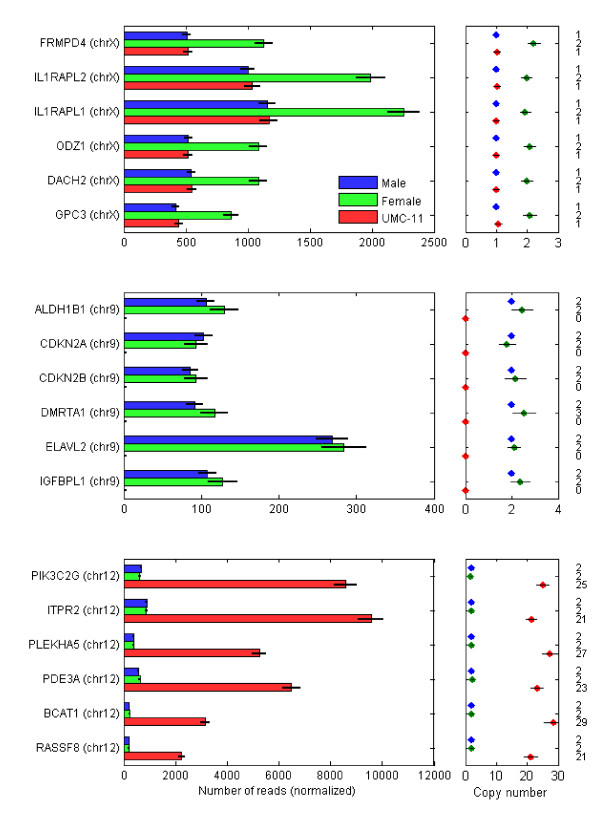
**Gene-centric copy number**. Top: the six genes with the most significant amplification in the female pool. Middle: the six genes with the most significant decreased copy number in the UMC-11 cells. Bottom: the six genes with the most significant amplification in the UMC-11 cells. The male pool is assumed to be diploid (autosomes) and haploid (allosomes).

The loci with the most significant p-values for higher copy number in the female pool are, not surprisingly, found on chromosome X (Figure 5A). In each case, this platform assayed the loci at copy number two in the female pool and one in the male pool and UMC-11 cells. The loci with the most significant lower copy number in UMC-11 cells are in the chromosome 9 deletion (Figure 5B), where the loci show copy number two in the female pool. The loci with the most significant amplifications are in the chromosome 12 amplification (Figure 5C), with copy number at almost 30.

Similarly, biological elements can be defined in terms of the coordinates of known DNA copy number polymorphisms [14]. By counting reads aligning within the coordinates of each established polymorphism, one can monitor population-variable copy number polymorphisms (not shown).

### Application 3: Mitochondrial copy number

**Figure 6:**
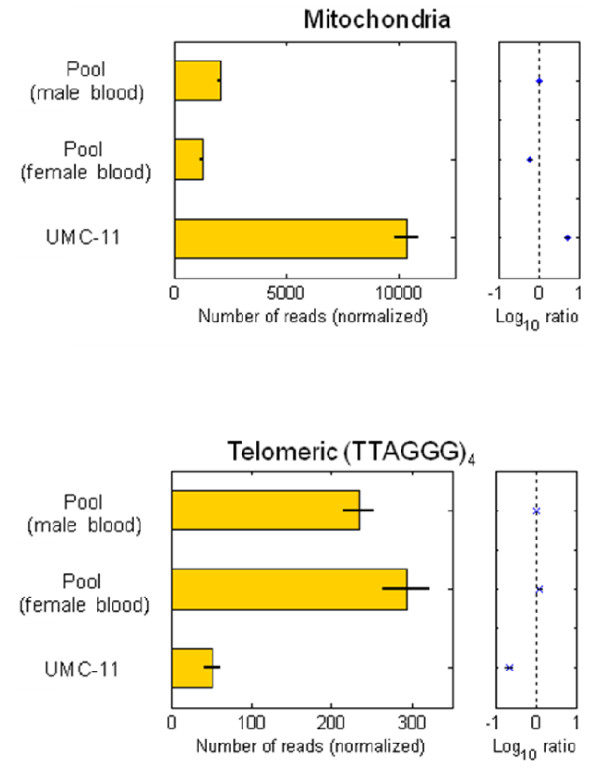
**Mitochondria and telomere copy number**.

Biological elements can also be defined as the mitochondrial DNA (mtDNA), in which case one assays the number of mitochondria in the cells. While the counts of mtDNA within each individual cell will vary, blood-derived cells on average contain fewer mitochondria relative to cell lines [15]. From the aligned sequence reads, we counted and normalized the number of reads aligning to mtDNA as a measure of the average mtDNA levels within each sample. Indeed, we found that the UMC-11 cells contain over 5 times more mitochondria than the blood-derived male and female pools (Figure 6A).

### Application 4: Telomere copy number

Finally, another fascinating biological element that can be interrogated is telomeric sequence. Telomeres protect the ends of chromosomes; are on average shorter in cells that have undergone many divisions, such as older cells, tumors, and cell lines; and comprise repetitive TTAGGG motifs [16]. We counted and normalized the number of sequence reads containing (TTAGGG)_4_. Strikingly, the UMC-11 cells do contain significantly fewer telomere-associated reads than either the female or male pools (Figure 6B).

## Discussion and Conclusions

Assaying DNA copy number by next-generation sequencing is robust and accurate. The method described here requires a simple genomic DNA library construction; returns integer copy number values for homogeneous cells; and has a large dynamic range. The platform is unbiased in the sense that genomic targets are not preselected, such as is the case with qPCR and microarrays, and thus, given a new genome assembly, a new set of copy number polymorphisms, or a new set of biological DNA elements, the sequence reads maintain utility through re-alignment.

Our research builds on previous efforts. The 'digital karyotyping' protocol uses restriction enzymes and SAGE sequencing technology to generate reads that have been used to measure copy number variation and detect infectious viral DNA[17,18]. Using Illumina deep-sequencing, fetal aneuploidy was assayed, identifying Down, Edward, and Patau syndromes based on chromosome-specific trisomy [19]. Illumina deep-sequencing has been used to examine copy number across nuclear chromosomes. Campbell et al[7] used paired-end reads from size-selected libraries to identify genomic structural rearrangements and, integrating estimates of copy number with paired-end reads mapping to distal locations, were able to identify breakpoint coordinates and novel DNA sutures. Recently, Chiang et al[8] sequenced a size-selected library and measured the log-ratio change between normal and tumor sample pairs across nuclear chromosomes. They elegantly show trade-off curves between read number, copy number change, and genomic resolution and show statistical determination of breakpoints. In comparing their results to array-based methods, they find the sequencing-based platform has a larger dynamic range. Yoon et al. [10] and Alkan et al. [9] developed similar methods for use in human resequencing projects, using over 1 billion reads and 30× genome coverage to identify copy number polymorphisms in disease-free cells.

The method described here expands on these groundbreaking studies in several ways. First, our library construction does not include a size selection and is thus lab automatable. Second, by using a single diploid reference sample along with a novel normalization algorithm, our method removes biases inherent in molecular biology protocols and outputs absolute copy number in addition to log-ratio values. Third, we defined an uncertainty that allows us to estimate upper and lower bounds and p-values for each copy number measurement, both for absolute and relative measurements. Forth, by defining biological elements as not only nuclear DNA blocks positioned evenly across the nuclear genome, this platform enables assaying other biologically meaningful DNA elements, including gene loci, known copy number polymorphisms, mitochondria, and telomeres.

Finally, these results were generated using an Illumina Genome Analyzer II instrument in June, 2008, with one sample per lane, resulting in only 3 to 7 million 36 nt reads per sample. Sequencing instrumentation continues to improve, allowing more reads at lower costs. As the resolution of this assay is inversely proportional to the number of aligned reads, sample multiplexing and increasing numbers of sequence reads will enable increased resolution and/or significantly decreased costs.

## Methods

### Proof of concept of the sequencing-based DNA copy number profiling assay

To develop and evaluate a sequencing based platform for assaying CNV, we prepared genomic DNA libraries from a pool of DNA derived from blood from non-diseased males; a pool of DNA derived from blood from non-diseased females; and DNA from the UMC-11 cell line, a lung carcinoid-derived cell line. DNA was fragmented using DNase. We did not test whether DNase can shear heterochromatin sequence such as in centromeres; however, we used only sequence reads that align uniquely to the genome and thus would not use sequence reads from regions of low sequence-complexity. The ends of the fragmented DNA were filled in or cleaved to produce blunt ends. This blunted DNA was ligated to adapters containing reverse complements on like adapters for suppression PCR as well as priming sites for a set of universal PCR primers and the Illumina sequencing primer. These "DNA Libraries" were then amplified using standard PCR conditions. We did not use size-selection after fragmentation, allowing lab-automation of library construction.

We sequenced libraries in the Illumina Genome Analyzer II sequencer, generating 6,744,152 (male pool), 3,283,370 (female pool), and 5,204,934 (UMC-11) reads (Table 2) (NCBI GEO accession GSE21159) Each 36 nt read contained a 3 nt molecular barcode for potential sample multiplexing which was trimmed before alignment.

**Table 2:** Sequencing reads (33 nt) and alignments.

Sample	Reads	Aligned to genome	%	Unambiguous match	%
**Female pool**	3,283,370	3,038,662	93%	2,061,012	63%
**Male pool**	6,744,152	6,193,928	92%	4,372,811	65%
**UMC-11**	5,204,934	4,768,159	92%	3,313,093	64%

### Genomic alignment

The resultant sequence reads were computationally aligned to the genome using the algorithm BWA [20]. We used only reads aligning to the genome with the highest (least ambiguous) score, minimizing incorrect mappings such as those from the female pool mapping to the Y chromosome. A shortcoming of the use of uniquely aligning reads is that reads aligning to sequence-identical segmental duplications within the HG18 genome will be discarded and these regions not monitored. Between 92% and 93% of the reads aligned to the genome, with between 63% and 65% aligning unambiguously (Table 2). For each unambiguously aligned read, we recorded the chromosome and 5' coordinate.

**Figure 7:**
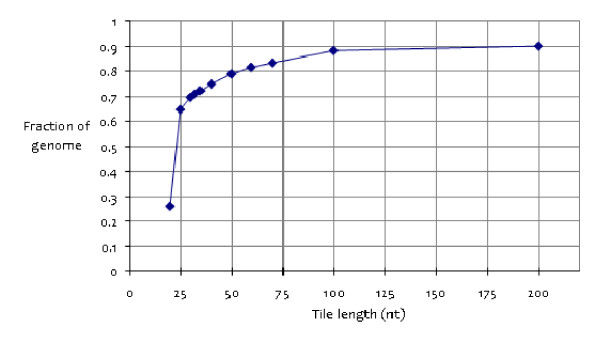
**Genome uniqueness at small scale lengths**. At each length scale, all oligos were aligned to the genome and unambiguously aligned reads were counted. The y-axis includes unsequenced regions (7%).

We computationally tested use of different read lengths. Shorter reads are more likely to align ambiguously whereas longer reads are more likely to align to a unique genomic location. We computationally generated all oligos (tiles) from the human genome at lengths from 20 nt to 200 nt. We aligned all to the genome and, for each tile length, determined the fraction of the reads that aligned to only one location (Figure 7). Seven percent of the human genome remains undetermined, containing repeat and low-complexity sequence patterns, e.g. centromeres.

Only 26% of the 20 nt tiles align uniquely, increasing to 65% at 25 nt, and to 70% at 30 nt, and smoothly increasing to 90% at 200 nt. A fundamental shift occurs between 20 and 25 nt: genome uniqueness changes from majority ambiguous to majority unambiguous. The 33 nt length (36 nt minus the 3 nt barcode) used in the study here represents an effective trade-off between sequencing costs (time and money) and read uniqueness.

### Counting & uncertainty

We selected biological elements for examination, such as DNA windows. For each window, we counted the number of reads aligning within the window. We compared the number to that found in a diploid reference sample. We used the DNA pooled from non-diseased males as a reference as the DNA should be diploid across autosomes and represent all chromosomes, including chromosome Y.

We defined an error model for a measurement "***x***" as a Poisson term from counting statistics:

With this error model, a block with 100 reads would have an uncertainty of ± 10. This error model reflects sampling uncertainty but neither biological or operator variability.

We used a uniform window size across the genome; however, a variable window size could be selected. The selected window size should reflect the total number of uniquely aligned reads available, the desired sensitivity/significance, and the sample cell-to-cell genomic heterogeneity. Here, we assumed that the cellular DNA was homogeneous and wished to have the power to distinguish copy number 3 from 2 at a p-value better than 0.001 assuming the described error model. This equates to 110 reads per genomic block. As we had 3.3 million reads from the female blood pool, of which 2.1 million uniquely aligned to the genome, and given the human genome of 3.1 billion base pairs, this results in a block size of 164 kb. We rounded to 150 kb blocks.

### Normalization

We normalized reads to account for the differing numbers of reads per sample and to determine absolute copy number. We found that the relative number of reads mapping to a DNA block depended on the molecular biology protocol used, thus necessitating use of a empirical normalization rather than simply a measure such as GC content. We used a male pool as a reference, and with this normalization were able to derive the copy number, upper and lower bounds, and the significance of any variation.

**Figure 8:**
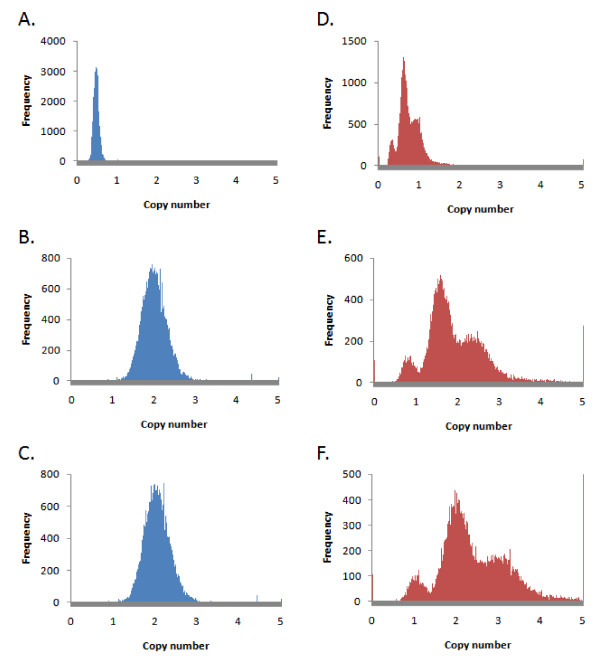
**Normalization allows absolute copy number estimate after normalization, assuming the male pool is diploid across all autosomes**. Left: female pool (blue). Right: UMC-11 (red). A and D: genome-wide distribution of the ratios of the number of reads aligning in each 150 kb window relative to the number in the same window from the male pool. B and E: counts are normalized by the total number of unambiguously aligned reads from each sample. C and F: counts are normalized by the mode from the distribution in B and E. Copy number of 1, 2, and 3 can be clearly seen in the UMC-11 sample.

We counted the reads mapping to each 150 kb block from the male and female pools and generated a ratio, female-to-male, for each block. There were roughly twice the number of reads for the male sample (Table 1), and thus the distribution of ratios peaks near 0.5 (Figure 8, top left). Normalizing by the number of aligned reads from the female and male samples results in a distribution peaked near one, as expected for samples with similar ploidy, and copy number two, assuming the male sample pool is diploid across autosomes (Figure 8, lower left).

The distribution of UMC-11 to male ratios after normalization by the number of reads shows multiple peaks, none of which are centered at an integer (Figure 8, middle right). We tried using the ratios to determine the normalization: using the ratio mean or the median failed to center a distribution peak at an integer. However, we were able to normalize the counts based on the mode of the ratio distribution across autosomes. After normalization by the ratio model, we find that the three peaks in the ratio distribution from the UMC-11 cell line are regions with one, two, and three copy number (Figure 8, lower right).

### Comparisons between samples

To compare CNV between samples, we calculated the significance of a difference using a t-test and p-value based on the normalized counts and normalized uncertainties. For each DNA element, the assay thus returns the absolute copy number, uncertainty, upper and lower bounds, and a p-value representing the significance of a measured difference.

### Validation with qPCR

We used qPCR (Taqman) to assay DNA copy number in UMC-11 cells. TaqMan primer-probe reagents were obtained through the Applied Biosystems Assays-by-Design custom assay service (Foster City, CA) and designed to fall outside of repeat regions.

## Competing interests

The authors declare that they have no competing interests.

## Authors' contributions

JCC and CKR designed the experiment. JCC, TX, RC, and KM analyzed the sequence read data. CKR, MB, HB, CDA (molecular biology) and SJ (IT) generated the data. JMJ provided guidance on the initial design, approved the project, and provided critical review of the project and manuscript. CAR coordinated and provided critical scientific input to the project and manuscript. JCC wrote the manuscript. All authors have approved the manuscript.

## Supplementary Material

Additional file 1 **Figure S1**. UMC-11 chromosome 9 copy number from the deep-sequencing platform (top) and Affymetrix SNP6 arrays and the Sanger Picnic algorithm [13] (bottom, green line). Click here for file

Additional file 2 **Figure S2**. UMC-11 chromosome 12 copy number from the deep-sequencing platform (top) and Affymetrix SNP6 arrays with the Sanger Picnic algorithm [13] (bottom, green line). Click here for file
