# Supplementary material for: DNA copy number, including telomeres and mitochondria, assayed using next-generation sequencing
Source: BMC Genomics. 2010 Apr 16;11:244. doi: 10.1186/1471-2164-11-244 (PMC2867831; doi:10.1186/1471-2164-11-244)

**Figure S1.** UMC-11 chromosome 9 copy number by deep-sequencing (top) and Affymetrix SNP6 array using the Sanger Picnic algorithm (bottom; green line).


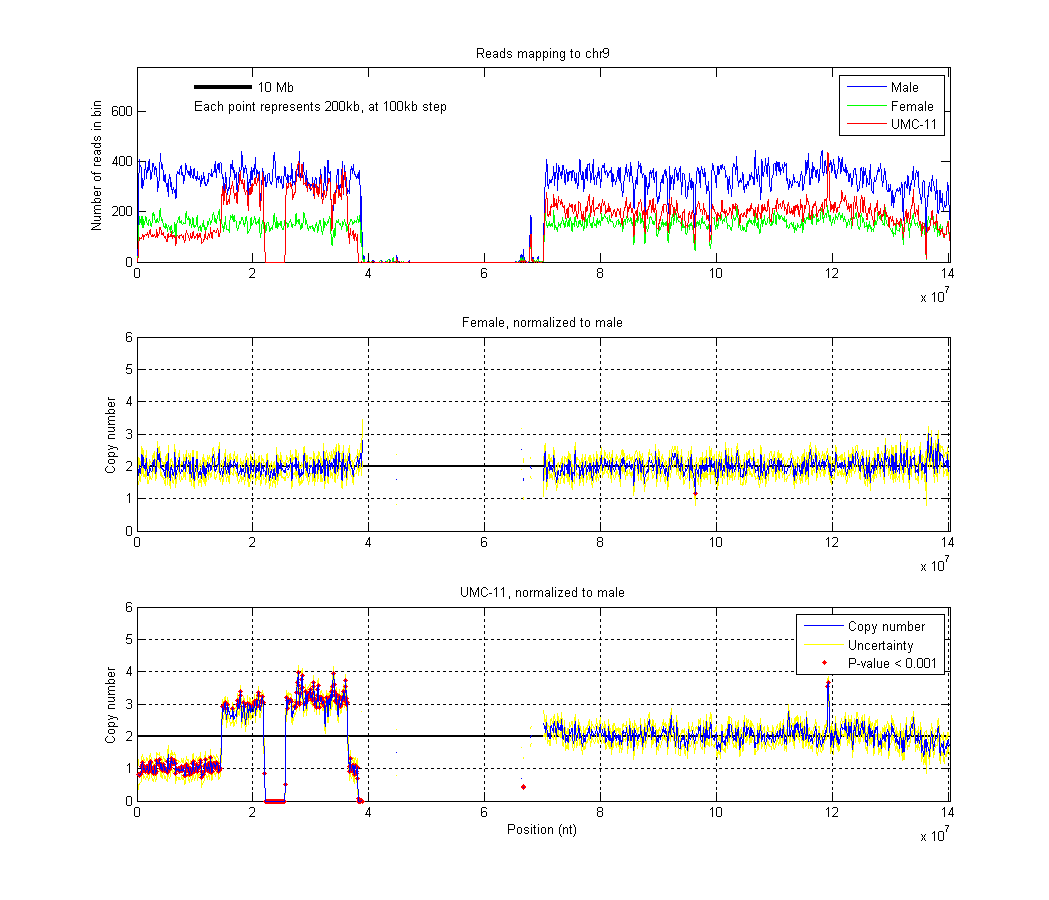

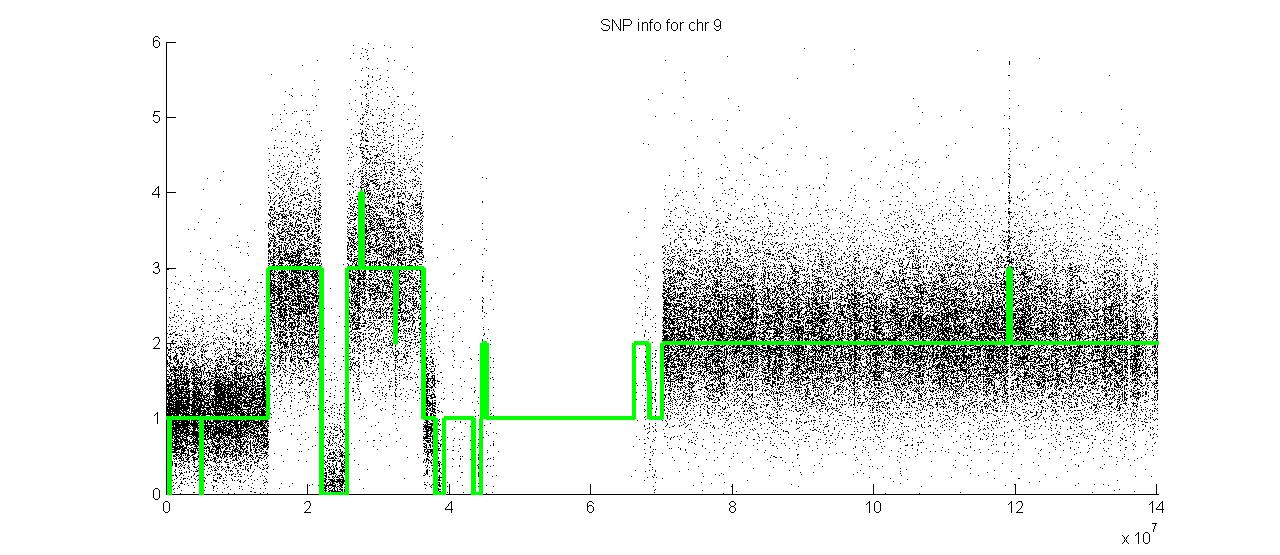

Supplement: Additional file 1 — Figure S1. UMC-11 chromosome 9 copy number from the deep-sequencing platform (top) and Affymetrix SNP6 arrays and the Sanger Picnic algorithm [13] (bottom, green line). [file 1471-2164-11-244-S1.DOC]
