# Supplementary material for: DNA copy number, including telomeres and mitochondria, assayed using next-generation sequencing
Source: BMC Genomics. 2010 Apr 16;11:244. doi: 10.1186/1471-2164-11-244 (PMC2867831; doi:10.1186/1471-2164-11-244)
Supplement: Additional file 2 — Figure S2. UMC-11 chromosome 12 copy number from the deep-sequencing platform (top) and Affymetrix SNP6 arrays with the Sanger Picnic algorithm [13] (bottom, green line). [file 1471-2164-11-244-S2.DOC]

**Figure S2.**  UMC-11 chromosome 12 CN by sequencing (top) and Affymetrix SNP6 array with the Sanger Picnic algorithm (bottom).


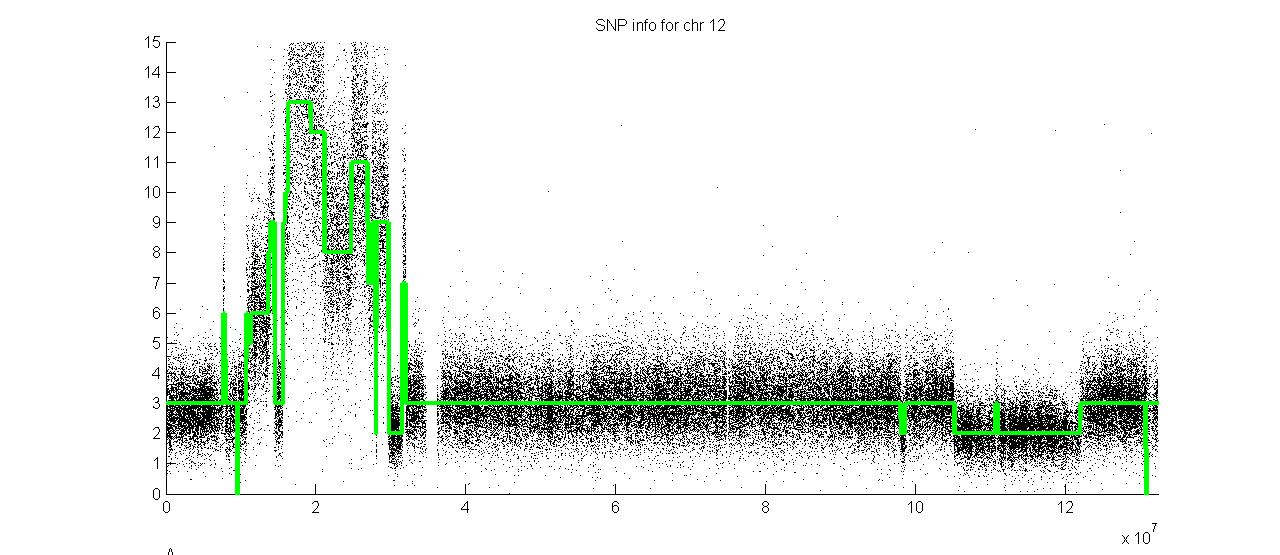

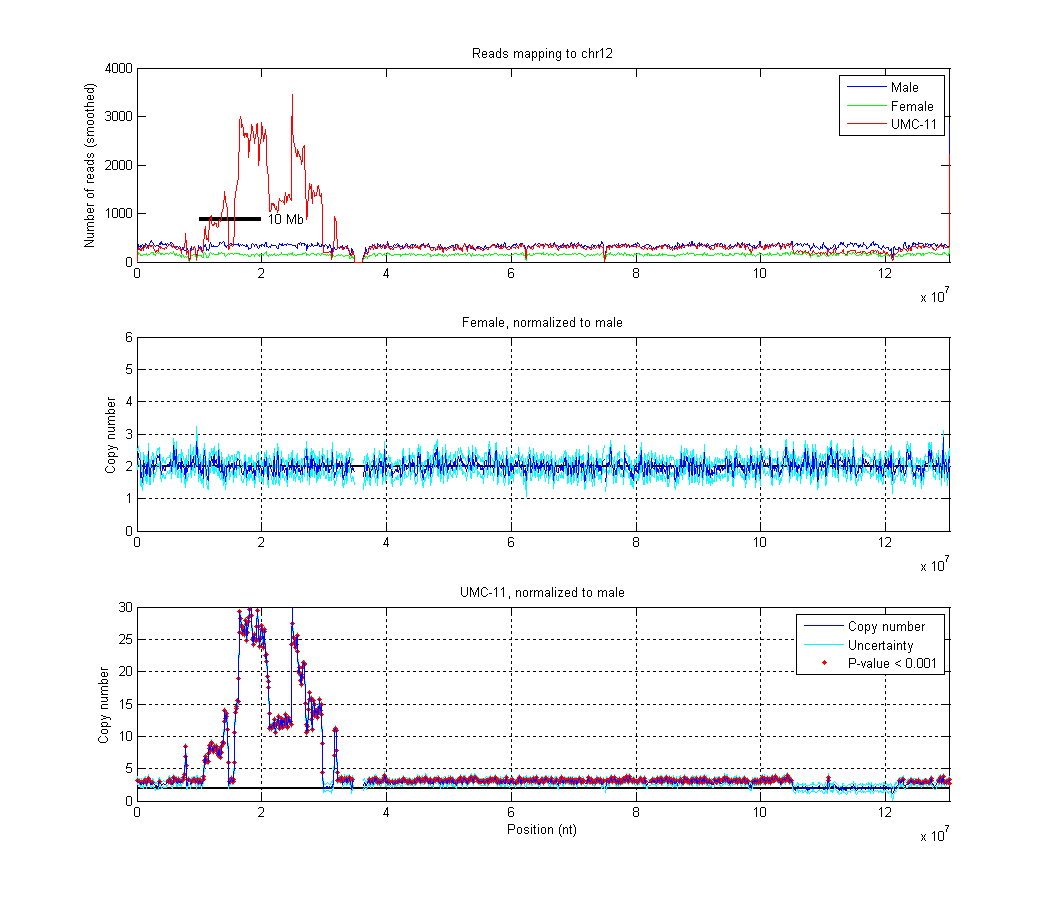


Output copy number = 26

Output copy number = 13
